# Expression of Estrogen Receptor- and Progesterone Receptor-Regulating MicroRNAs in Breast Cancer

**DOI:** 10.3390/genes12040582

**Published:** 2021-04-16

**Authors:** Tatiana Kalinina, Vladislav Kononchuk, Efim Alekseenok, Darya Obukhova, Sergey Sidorov, Dmitry Strunkin, Lyudmila Gulyaeva

**Affiliations:** 1 Institute of Molecular Biology and Biophysics, Federal Research Center of Fundamental and Translational Medicine, Timakova Str. 2/12, 630117 Novosibirsk, Russia; kononchuk@niimbb.ru (V.K.); alekseenok@niimbb.ru (E.A.); obukhovada555@gmail.com (D.O.); strunkind@mail.ru (D.S.); gulyaeva@niimbb.ru (L.G.); 2 Department of Breast Pathology, Novosibirsk Municipal Publicly Funded Healthcare Institution Municipal Clinical Hospital #1, Zalessky Str. 6, 630047 Novosibirsk, Russia; svsidorov@yandex.ru

**Keywords:** microRNA, breast cancer, biomarker, lymph node metastasis, hormone-dependent carcinogenesis

## Abstract

In ~70% of breast cancer (BC) cases, estrogen and progesterone receptors (ER and PR) are overexpressed, which can change during tumor progression. Expression changes of these receptors during cancer initiation and progression can be caused by alterations in microRNA (miR, miRNA) expression. To assess the association of BC progression with aberrant expression of miRNAs that target ER and PR mRNAs, we quantified miR-19b, -222, -22, -378a, and -181a in BC samples (*n* = 174) by real-time PCR. Underexpression of miR-222 and miR-378a in stage T2–T4 BC was characteristic for HER2-overexpressing tumors. In addition, the expression of miR-181a and miR-378a was higher in these tumors than in tumors with a HER2 IHC score of 0 or 1+. In tumors with a Ki-67 index ≥ 14%, all tested miRNAs were underexpressed in BC with a high Allred PR score (6–8). In ER-and-PR–negative tumors, miR-22, miR-222, miR-181a, and miR-378a underexpression was associated with Ki-67 index > 35% (median value). MiR-19b and miR-22 underexpression could be a marker of lymph node metastasis in ER- and/or PR-positive tumors with HER2 IHC score 0. Thus, the association of miR-19b, miR-22, miR-222, miR-378a, and miR-181a levels with BC characteristics is influenced by the status of tumor ER, PR, HER2, and Ki-67.

## 1. Introduction

Despite modern advances in medicine, cancer remains one of the leading causes of death [1]. In women, the most prevalent malignant tumor is breast cancer (BC). BC treatment depends on expression levels of estrogen receptor (ER), progesterone receptor (PR) and HER2 (a receptor for Epidermal Growth Factor, EGF). Approximately 70% of cases of this cancer are characterized by increased expression of ER and PR, which are involved in breast carcinogenesis [2]. Enhanced expression of their target genes (e.g., *CCND1*, *VEGF*, and *MYC*) promotes tumor growth through the regulation of the cell cycle, signaling, differentiation, and apoptosis [3,4,5]. Nonetheless, ER and PR status may change during tumor progression to metastasis. The change of ER or PR status from positive to negative is associated with a worse prognosis [6]. Therefore, a study of the pathways involved in the regulation of ER and PR expression may help the understanding of the mechanisms of cancer progression and evolution.

One of the factors affecting ER and PR expression during the initiation and progression of cancer is a change in the microRNA (miR, miRNA) expression profile [7]. These short single-stranded RNAs participate in post-transcriptional regulation of the expression of various genes by acting as oncogenic or tumor-suppressive molecules. A number of miRNAs target ER and PR; therefore, it can be assumed that their expression also changes during BC initiation and progression.

In this study, to assess the association of aberrant expression of miRNAs that target ER and PR mRNAs with the progression of BC, we quantified miR-19b, -222, -22, -378a, and -181a in BC samples (*n* = 174). We demonstrated that the expression of these miRNAs may correlate with the expression of PR and HER2, and in certain cases, it correlates with T and N stages and Ki-67 index, suggesting that these miRNAs may serve as biomarkers.

## 2. Materials and Methods

### 2.1. Tissue Samples

A total of 174 pairs of BC tissue samples and samples of normal adjacent tissue from female patients who had not received preoperative pharmacotherapy, were collected in 2017–2018 at Novosibirsk municipal publicly funded healthcare institution Municipal Clinical Hospital #1. Tissue samples were placed in an RNAlater™ Stabilization Solution (Invitrogen™, Carlsbad, CA, USA) and kept at −20 °C until experiments were performed. Clinicopathologic information was obtained by reviewing medical records and reports on results of immunohistochemical assays. The following variables were determined: the T stage, N stage; immunohistochemical (IHC) scores on ER, PR, HER2, and Ki-67 (Table 1). For cases with HER2 IHC-score 2+, the determination of the final HER2 status was carried out using FISH. All patients recruited into the study had grade 2 (G2) tumors.

### 2.2. MicroRNA Isolation

Total miRNA was extracted from human tissue by a previously published protocol [9].

### 2.3. MiRNA Reverse Transcription and Real-Time PCR (RT-PCR)

Relative expression levels for miRNAs were measured by real-time reverse transcription-PCR. A reverse-transcription reaction was carried out using stem-loop primers [10] and the RT-M-MuLV-RH kit (Biolabmix Ltd., Novosibirsk, Russia). Real-time PCR was performed with TaqMan probes and the BioMaster UDG HS-qPCR (2×) kit (Biolabmix Ltd.). To detect PCR products, a CFX96™ Detection System (Bio-Rad Laboratories, Hercules, CA, USA) was applied. Small nuclear RNAs U44 and U48 were used to normalize the data.

Primers for the reverse transcription were as follows: hsa-miR-19b-3p, 5′- GTC GTA TCC AGT GCA GGG TCC GAG GTA TTC GCA CTG GAT ACG ACT CAG TTT -3′; hsa-miR-22-3p, 5′- GTC GTA TCC AGT GCA GGG TCC GAG GTA TTC GCA CTG GAT ACG ACA CAG TTC T -3′; hsa-miR-222-3p, 5′- GTC GTA TCC AGT GCA GGG TCC GAG GTA TTC GCA CTG GAT ACG ACA CCC AGT A -3′; hsa-miR-378a-3p, 5′- GTC GTA TCC AGT GCA GGG TCC GAG GTA TTC GCA CTG GAT ACG ACG CCT TCT -3′; hsa-miR-181a-5p, 5′- GTC GTA TCC AGT GCA GGG TCC GAG GTA TTC GCA CTG GAT ACG ACA CTC ACC G -3′; U44, 5′- GTC GTA TCC AGT GCA GGG TCC GAG GTA TTC GCA CTG GAT ACG ACA GTC AGT T -3′; U48, 5′- GTC GTA TCC AGT GCA GGG TCC GAG GTA TTC GCA CTG GAT ACG AGA CGG TCA G-3′.

The following specific oligonucleotides were employed for RT-PCR: hsa-miR-19b-3p, (forward primer) 5′- GCCGTGTGCAAATCCATGCA -3′, (probe) 5′-(R6G)-TTCGCACTGGATACGACTCAGTTT-(BHQ1)-3′; hsa-miR-22-3p, (forward primer) 5′-GCCGAAGCTGCCAGTTGA-3′, (probe) 5′-(R6G)-TTCGCACTGGATACGACACAGTTCT-(BHQ1)-3′; hsa-miR-222-3p, (forward primer) 5′-GCCGCAGCTACATCTGGC-3′, (probe) 5′-(R6G)-TTCGCACTGGATACGACACCCAGTA-(BHQ1)-3′; hsa-miR-378a-3p, (forward primer) 5′-GCCGCACTGGACTTGGAGTC-3′, (probe) 5′-(R6G)- TTCGCACTGGATACGACGCCTTCT -(BHQ1)-3′; hsa-miR-181a-5p, (forward primer) 5′-GCCGCAACATTCAACGCTGT-3′, (probe) 5′-(R6G)-TTCGCACTGGATACGACACTCACCG-(BHQ1)-3′; U44, (forward primer) 5′- GCCGCTCTTAATTAGCTCT-3′, (probe) 5′-(R6G)-TTCGCACTGGATACGACAGTCAGTT-(BHQ1)-3′; U48, (forward primer) 5′-CCCTGAGTGTGTCGCTGATG-3′, (probe) 5′-(R6G)-TTCGCACTGGATACGAGACGGTCAG-(BHQ1)-3′. A similar type of reverse primer targeting the stem-loop region in the synthesized cDNAs was 5′-AGTGCAGGGTCCGAGGTA-3′. Each sample was analyzed in triplicate. Relative expression level was assessed based on threshold cycle (Ct) values taking into account PCR efficacy (E) for both the analyzed and reference RNAs.

### 2.4. Statistical Analysis

STATISTICA software (version 12; TIBCO Software Inc., Palo Alto, CA, USA) was used for statistical data analysis and plotting. Data are presented as median values. The Shapiro–Wilk test was used to check data normality. Since the distribution was not normal in some groups, the statistical analysis was carried out using the non-parametric Mann–Whitney U test. Data with *p* < 0.05 were regarded as statistically significant.

## 3. Results

### 3.1. Analysis of miR-19b, miR-22, miR-222, miR-378a, miR-181a Expression in Breast Cancer

According to the miRTarBase database [11], PR expression in humans can be regulated by miR-181a and miR-378a (strong evidence). In addition, using a reporter assay, western blot analysis and qPCR, it was shown that the ER is a target for miR-206, miR-18a/18b, miR-22, miR-19a/19b, miR-20b, and miR-221/222 (Table 2). First, we were interested in miRNAs, which can target both receptors with a high probability. We chose miR-181a, miR-378a, miR-22, miR-222, and miR-19b. In addition to the fact that these miRNAs are likely to target both receptors, their expression level has been shown in few studies to be inversely correlated with ER status. A large number of target genes are known for these miRNAs, which can play the role of oncogenes or tumor suppressors in BC. All of the above points to the important role of these miRNAs in BC.

**Figure 1:**
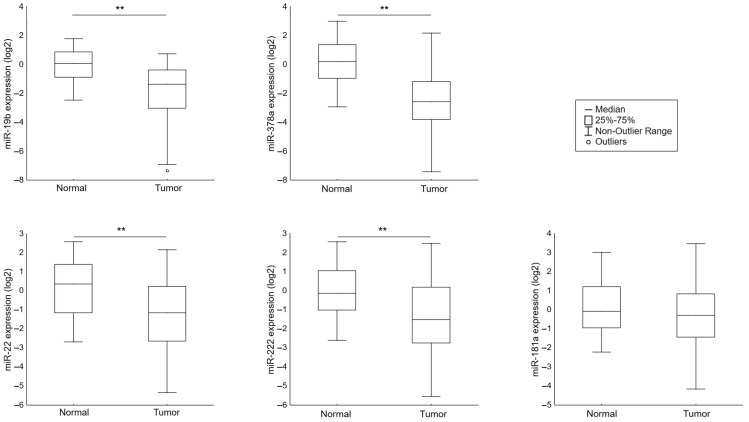
The comparison of microRNA expression between normal and cancerous tissue groups. The Y axis presents the expression level ($\log_{2}2^{–\Delta \mathrm{Ct}}$). ** *p* < 0.0001.

The relative levels of selected miRNAs were determined in 174 pairs of tumor and healthy tissues by RT-PCR (Figure 1). We found that the expression of miR-19b, miR-378a, miR-222, and miR-22 was significantly reduced in tumor tissues.

### 3.2. Analysis of the Association of MiR-19b, MiR-22, MiR-222, MiR-378a, and MiR-181a Expression with ER, PR, HER2 Status and Ki-67 Index

We investigated whether the expression of a number of ER-, and PR-associated miRNAs depends on the status of ER, PR, HER2, Ki-67 index, and age. So far, several studies have shown that ER is a target for miR-19b, miR-22 and miR-222 [29,32,33], whereas miR-181a targets the PR mRNA [34]. Regulation of PR expression by miR-378a has been proved by means of pig granulosa cells as a model [35].

We observed that miR-22 was underexpressed in tumors of patients with a high Ki-67 index (Table 3). The expression levels of miR-378a and miR-181a were significantly higher in tumors of patients with HER2-overexpressing/amplified cancer than in tumors with HER2 0 and HER2 1+ scores. Expression of miR-22 and miR-378a was significantly lower in tumors of patients older than 50 years compared to tumors of younger patients. No association with the presence or absence of ER and PR expression in tumors was found.

### 3.3. Expression of MiR-19b, MiR-22, MiR-222, MiR-378a, and MiR-181a in Relation to Clinicopathologic Features of ER- and/or PR-Positive BC and ER-and-PR-Negative BC

Next, we evaluated the relation between the expression of miRNAs and clinicopathologic features of tumors with various ER and PR status (Table 4). We noted that the expression of miR-222 was significantly lower (3-fold) in samples of ER- and/or PR-positive tumors from patients with BC stages T2–T4. We also divided patients into subgroups based on their Ki-67 index. In ER-and-PR-negative BC, an association of miR-22, miR-222, miR-378a, and miR-181 expression with the Ki-67 was observed. The expression of these miRNAs was significantly lower in the samples where the Ki-67 index was higher than the median value (>35%) as compared to cases with the lower Ki-67. The most significant difference was found for miR-181a.

### 3.4. Expression of MiR-19b, MiR-22, MiR-222, MiR-378a, MiR-181a in Relation to Clinicopathologic Features of HER2-Positive BC or HER2-Negative BC

Another important characteristic that is used for tumor subtyping is HER2 status. We assessed the relation between levels of the miRNAs and clinicopathologic features of HER2-positive or -negative BC (Table 5).

The amount of all miRNAs turned out to be associated with the T stage in HER2-expressing tumors. MiRNA levels were lower in patients with BC stages T2–T4. The most significant differences (*p* < 0.01) were observed for miR-222 and miR-378a. The levels of miR-222, miR-378a, and miR-181a correlated with the level of expression of HER2. The levels of these miRNAs were higher in tumor tissues with high HER2 expression (a score of 2–3 according to IHC) as compared to tumors with low HER2 expression (1 ICH score). For miR-181a, a relation with PR expression was also revealed. The amount of this miRNA was significantly higher in BC tissues with low PR expression (a score of 0–5 in the IHC assay, Allred scoring method).

For tumors not expressing HER2, an association of miR-22 and miR-19b levels with the presence of lymph node metastasis (LNM) was detected. The level of these miRNAs was significantly lower in tumors of patients with LNM as compared to cases without. Because there was a relatively small number of metastatic cases among patients with ER-, PR-, and HER2-negative BC (four patients) in our study population, we also quantified miRNAs separately for HER2-negative ER- and/or PR-positive tumors. The lower miR-19b level in LNM cases was indeed more statistically significant for such tumors.

Here, we assigned patients with HER2-low tumors (i.e., 1+ and lack of *ERBB2* amplification) to the same group as patients in whom HER2 gene amplification was detected. This was because the level of HER2 expression is higher in HER2 1+ BC compared to HER2 0 tumors [36]. In addition, these variants of tumors differ in the expression profile of a number of genes, and clinically, HER2-low BC shows more axillary lymph-node involvement compared to HER2 0 BC [37]. Thus, biologically HER2 1+ tumors differ significantly from HER2 0 tumors. However, when choosing a treatment, only patients with HER2 IHC score 3+ or HER2 IHC score 2+ (positive for HER2 amplification by *in situ* hybridization, ISH) are classified as HER2-positive type. Therefore, we also analyzed miRNA expression separately for patients with HER2 0 and HER2-low tumors and for patients with HER2-overexpressing/amplified tumors (Table 6). The association with tumor size in patients with HER2-overexpressing/amplified BC was observed only for miR-222 and miR-378a. In patients with HER2 0 and HER2 1+ BC, the level of miR-19b was significantly reduced in cases with metastasis. When we divided patients into such groups, no association of miRNAs with the PR level was found.

No association was detected between miRNA expression and the Ki-67 index when we subdivided the patients into groups according to HER2 status.

### 3.5. Expression of MiR-19b, MiR-22, MiR-222, MiR-378a, and MiR-181a in Relation to Clinicopathologic Features of Tumors with the Ki-67 < 14% or ≥ 14%.

Yet another characteristic that underlies BC subtyping is the Ki-67 index. We found miR-222 to be significantly downregulated in patients with BC stage T2–T4 when the Ki-67 was ≥ 14% (Table 7). In addition, in patients with the Ki-67 index ≥ 14%, all miRNA amounts were associated with PR expression; the amounts of miRNAs were higher in tumors with low PR expression (a score of 0–5) compared to tumors with higher PR expression (a score of 6–8).

## 4. Discussion

It is generally recognized that miRNAs play an essential role in carcinogenesis. By altering the expression of genes involved in the regulation of the cell cycle, apoptosis, epithelial–mesenchymal transition, and/or intercellular signaling, miRNAs can serve as oncogenic or tumor-suppressive molecules [38]. Recent studies showed that certain miRNA expression profiles correlate with tumor aggressiveness, treatment responses, and treatment outcomes, suggesting that miRNAs can be used as diagnostic or prognostic markers [39].

BC is the most common cancer in women. Efforts have been made to search for miRNAs whose changes in expression are critical for initiation and/or progression of BC. Nonetheless, most of these studies are conducted without considering the tumor subtype. On the other hand, changes in the expression and activity of ER and PR are associated with the initiation and progression of BC. In this regard, it can be expected that these disorders are accompanied by a change in the expression of miRNAs targeting steroid receptors. For instance, it is known that ER mRNA is a target for miR-19b, miR-22, and miR-222, whose relationship with breast carcinogenesis has already been demonstrated, whereas PR mRNA is a target for miR-378a and miR-181a.

The choice of BC treatment is based on the status of ER, PR, HER2, and Ki-67 index. The expression levels of these receptors and Ki-67 can, among other things, lead to variations in the expression of miRNAs. Therefore, here we divided all patients into groups depending on the ER and PR status, on the HER2 status, and on the Ki-67 index. In practice, when choosing a method of treatment, only cases with a HER2 IHC score of 3+ or HER2 IHC score of 2+ (with HER2 amplification) are classified as HER2-positive tumors. However, biologically HER2 1+ tumors differ significantly from HER2 0 tumors [36]. HER2-low tumors (1+ and 2+ with lack of HER2 amplification) and HER2 0 tumors differ in the expression profile of a number of genes, and HER2-low tumors are characterized by larger tumor sizes and more nodal involvement compared to HER2 0 tumors [37]. Therefore, we also analyzed the levels of miRNA expression separately for the group of patients with HER2 1+, 2+, 3+ tumors, for the group of patients with HER2 0 tumors, for the group of patients with HER2-overexpressing/amplified tumors, and for the group of patients with HER2 0, 1+ tumors. The main identified correlations between differences in miRNA expression and tumor characteristics in these groups are presented in Table 8.

It is reported that miR-22 and miR-378a play a tumor suppressor role in BC [14,16,40]. The results of our study are consistent with the above studies. We showed that miR-22 expression is lower in tumors with the Ki-67 ≥ 14% relative to tumors with the lower Ki-67. The expression of miR-378a turned out to be associated with that of Ki-67 in ER-and-PR-negative tumors. Its level proved to be significantly lower in samples with the Ki-67 > 35%. In our group of HER2 0 breast tumors, miR-22 was downregulated in the samples from BC patients who have LNM. However, when we combined patients with HER2 0 and HER2 1+ tumors into one group, the association of miR-22 with metastases was not significant. Thus, the level of miR-22 can be a marker of LNM only for patients with HER2 IHC-score 0.

The expression of miR-378a and miR-22 was detected to be related to the age of the patients: the level of miRNAs is significantly lower in tumors of patients over 50 years old. The median age at menopause among women from industrialized countries ranges between 50 and 52 years [41]. Thus, it can be expected that changes in the expression of these miRNAs may be associated with menopause in women.

It is known that miR-222 usually plays an oncogenic role in carcinogenesis [19,42]. By contrast, here we revealed that miR-222 underexpression is associated with a large tumor size (stage T2–T4) in the group of tumors with ER- and/or PR-positive status, in the group of HER2-positive tumors, and in the group of tumors with the Ki-67 ≥ 14%. Additionally, the level of this miRNA is lower in the tumor samples with the Ki-67 ≥ 35% in the ER-and-PR-negative group. It should be noted that according to the TargetScan database, mRNA of the gene encoding Ki-67 is predicted as a target for miR-222 [43]. That is, a high level of Ki-67 may be a consequence of, among other things, a decrease in the expression of this miRNA.

For miR-19b and miR-181a, there is conflicting evidence indicating both oncogenic and tumor-suppressive functions of these miRNAs in BC [23,44,45]. We showed that miR-19b is downregulated in ER- and/or PR-positive HER2-negative BC with LNM. The amount of miR-181a is significantly lower in the tumor samples with the Ki-67 ≥ 35% in the ER-and-PR-negative group.

In the group of tumors with HER2 IHC scores 1+, 2+, 3+, the levels of all studied miRNAs are lower in stage T2–T4 BC as compared to tumors with stage T1. However, the association with tumor size in HER2-overexpressing/amplified BC is retained only for miR-222 and miR-378a. It is noteworthy that among the potential targets of miRNAs (according to the TargetScan database) are HER3 mRNA (the target of miR-22, miR-222, miR-19), which is a partner of HER2, and CCR4 mRNA (the target of miR-22, miR-222, miR-378a), whose expression has been reported previously to correlate with HER2 status [46]. Both proteins are known to take part in cancer progression. Among the predicted targets of miRNAs, there are also MAPK1 (the target of all five microRNAs), AKT2 (the target of miR-181, miR-222, miR-22), PIK3CA (the target of miR-19, miR-222, miR-378a,), and CDK6 (the target of miR-222, miR-378a, miR-19, miR-181), which encode participants in the signaling pathways regulated by HER2 [47,48,49]. Thus, it is possible that these miRNAs play a tumor-suppressing role in patients with smaller tumors through participation in the regulation of the expression of these genes.

We also demonstrated that in the group of tumors with the Ki-67 index ≥ 14%, miRNAs levels were higher in tumors with lower PR expression (a score of 0–5 in the IHC assay, Allred scoring method). The regulation of PR expression by miR-181a and miR-378a has already been demonstrated previously. Nevertheless, the receptor’s mRNA is also predicted to be a target for miR-19b, miR-22, and miR-222 (Table 2). Thus, changes in PR expression during breast carcinogenesis can be caused by alterations in the levels of these miRNAs. We also found that miR-222, miR-378a, and miR-181a are upregulated in HER2-overexpressing tumors as compared to cases with a HER2 expression score of 1+. The difference is most significant for the miR-378a. It has been previously demonstrated that the expression of miR-378 is regulated by the HER2 signaling pathway [50]. In addition, according to the TargetScan database, HER2 mRNA is a potential target for miR-378a. The increase in this miRNA expression is possibly associated with a cellular response to HER2 upregulation.

## 5. Conclusions

Our study indicates that the association of miR-19b, miR-22, miR-222, miR-378a, and miR-181a levels with the characteristics of breast tumors is influenced by the status of ER, PR, HER2, and the Ki-67 index. Underexpression of these miRNAs in stage T2–T4 BC is characteristic only for HER2-expressing tumors (IHC scores 1+, 2+, 3+). In the same tumors, the expression of miR-222, miR-181a, and miR-378a is stronger when the level of the HER2 protein is high (IHC scores 2+, 3+). In tumors with the Ki-67 ≥ 14%, the expression of all tested miRNAs is low when PR expression is high (a score of 6–8 in IHC analysis). In ER- and PR-negative tumors, underexpression of miR-22, miR-222, miR-181a, and miR-378a is associated with a high Ki-67 index (>35%). We also discovered that decreased levels of miR-19b and miR-22 may be a marker of LNM in ER- and/or PR-positive tumors with HER2 IHC score 0.

## Figures and Tables

**Table 1:** Characteristics of the breast tumors under study.

Characteristics		ER- And/Or PR-Positive (*n* = 130)	ER-and-PR-Negative (*n* = 44)	HER2 2+, 3+ (*n* = 43)	HER2 1+ (*n* = 31)	HER2 0 (*n* = 100)	Triple-Negative (*n* = 20)
Age (mean and range, year)		60 (27–90)	52 (38–76)	56 (39–90)	57 (44–84)	58 (27–83)	54 (38–76)
T stage	T1	46	18	16	8	40	9
	T2	77	23	22	22	56	10
	T3	2	1	3	-	-	-
	T4	5	2	2	1	4	1
N stage	N0	70	29	27	11	61	16
	N1	46	8	10	15	29	3
	N2	10	6	5	3	8	1
	N3	4	1	1	2	2	-
Ki-67 index	<14%	73	7	14	14	52	3
	14–39%	47	19	20	13	33	8
	≥40%	10	18	9	4	15	9
ER score	0–2	4	44	18	7	23	20
	3–5	13	-	2	1	10	-
	6–8	113	-	23	23	67	-
PR score	0–2	22	44	24	13	29	20
	3–5	34	-	7	3	24	-
	6–8	74	-	12	15	47	-
HER2 score	0	80	20	-		100	20
	1	23	8	-	31	-	-
	2–3	27	16	43	-	-	-

Estrogen and progesterone receptors (ER and PR). ER and PR were graded by the Allred scoring method [8].

**Table 2:** The miRNAs regulating the expression of ER and PR.

miRNA	Target (Score Class *)	Other Targets Associated with BC **	Association with ER or PR Status	Association with Tumor Characteristics
hsa-miR-181a-5p	**PR**; ER (very high)	NLK, GATA6, BCL2, ATM, HIPK2, HRAS, SIRT1, DUSP5, FOS, MTMR3, XIAP	The averages of the expression fold change of miR-181a was significantly lower in the PR-positive group compared to the PR-negative group of BC patients [12].	MiRNA was downregulated in lymph node metastasis group of BC patients [13].
hsa-miR-378a-3p	**PR**; ER (medium)	VEGFA, NPNT, MYC, CYP19A1	Not available, but miR-378a-3p expression was down-regulated in tamoxifen-resistant MCF-7 cells [14].	Lower expression levels of miR-378a-3p were associated with poor prognosis for tamoxifen-treated patients [14].
hsa-miR-22-3p	**ER**; PR (high)	BMP7, MYCBP, RGS2, NCOA1, ERBB3, PTEN, SP1	ER-positive breast tumor specimens had significantly lower miR-22 levels than ER-negative specimens [15].	Downregulation of miR-22 was significantly associated with the poor differentiation, advanced clinical stage, as well as lymphatic and distant metastasis in breast cancer [16].
hsa-miR-222-3p	**ER**; PR (high)	STAT5A, MMP1, FOXO3, CDKN1C, FOS, ICAM1, PTEN, ETS1, RECK, TRPS1, CERS2, DKK2	MiR-221/222 was more abundant in triple-negative tumors than in ER/PR–positive tumors [17]. MiR-222, but not miR-221, led to suppress the expression of the luciferase reporter plasmid carrying the full-length 3′-UTR of ESR1 mRNA signal by more than 40% [15].	Increased expression of miR-222 was associated with lymph node positive status [18]. High expression levels of miR-222 in patients with ER^+^ breast cancer were associated with decreased disease-free survival as compared to ER^+^ breast cancer patients exhibiting low levels of miR-222 [19].
hsa-miR-221-3p		FOXO3, CDKN1C, TBK1, DKK2, BRAP, USP18, ICAM1, FOS, BNIP3, PTEN, ETS1, TRPS1, CERS2, STAT5A, RECK	Expression level of miR-221 was significantly lower in ERa-negative than in ERa-positive tumors [20].	Low miR-221-3p expression may contribute to the poor outcome of triple negative BC patients [21].
hsa-miR-19b-3p	**ER**; PR (high)	PTEN, MYLIP, SOCS1, TLR2, CYP19A1, TGFBR2, SMAD4, MYCN	The expression of both miR-19a and b in BC tissue samples with ER-positive status were down-regulated compared to those with ER-negative. The most significant difference was for the MiR-19b [22].	The miR-19b expression was associated with distant metastasis and TNM stage [23]. MiR-19b-1 stable overexpression in MDA-MB-231 caused tumor growth arresting entirely [24].
hsa-miR-19a-3p		HOXA5, MECP2, PTEN, CCND1, ERBB4, SOCS1, TLR2, TGFBR2, SMAD4, TNF, ABCA1, MYCN		High serum miR-19a levels are associated with inflammatory breast cancer [25].
hsa-miR-206	**ER**; PR (very high)	NOTCH3, CCND2, CDK4, TKT, CCND1	The expression of miR-206 was inversely correlated with ER mRNA level in breast cancer tissues [26].	Decreased miR-206 was significantly associated with advanced clinical stage and lymph node metastasis [27].
hsa-miR-20b-5p	**ER**; PR (high)	CDKN1A, MYLIP, VEGFA, EPHB4, LIMK1, PTEN	-	High expression of miR-20b associated with breast cancer brain metastasis [28].
hsa-miR-18a-5p	**ER**; PR (medium)	PTEN, TNFSF11, NR3C1, HIF1A, ATM, TGFBR2, SMAD4, RUNX1, CTGF	Pre-miR-18a levels were significantly higher in ER-positive tumors compared with negative tumors [29]. Expression levels of miR-18a were strongly and negatively correlated with PR protein scores. MiR-18a expression was much higher in ER-negative than in ER-positive tumors [20].	A relatively high miR-18a expression was associated with a poor prognosis, especially in the luminal subtype [30].
hsa-miR-18b-5p		MDM2, CTGF	MiR-18b expression was not correlated with ERa protein expression [20].	Low miR-18b expression was significantly associated with improved survival in HER2-negative breast cancer [20].

The receptor, which is a target for microRNA according to the miRTarBase database, is highlighted in bold. * The score class for the predicted target receptor is given according to the mirDIP database [31]. ** miRTarBase database data.

**Table 3:** Associations between the amounts of miR-19b, miR-22, miR-222, miR-378a, or miR-181a in tissue samples from BC patients and ER, PR, HER2 status, Ki-67 index, or age.

Characteristics		*n*	Relative Level * of miRNA and *p*-Value									
			miR-19b	*p*-Value	miR-22	*p*-Value	miR-222	*p*-Value	miR-378a	*p*-Value	miR-181a	*p*-Value
ER and PR status	ER^+^ and/or PR^+^	130	0.48	0.974	0.46	0.533	0.41	0.535	0.20	0.407	0.85	0.518
	ER^−^ and PR^−^	44	0.46		0.57		0.54		0.24		0.97	
HER2 status	HER2^+^	43	0.55	0.345	0.48	0.470	0.70	0.181	0.44	**0.013**	1.33	**0.015**
	HER2^−^	131	0.42		0.48		0.41		0.18		0.74	
Ki-67 index (%)	<14	80	0.52	0.335	0.76	**0.043**	0.46	0.537	0.24	0.407	0.85	0.904
	≥14	94	0.38		0.36		0.43		0.19		0.96	
Age	≤50	58	0.59	0.238	0.97	**0.002**	0.54	0.283	0.36	**0.003**	1.24	0.133
	>50	116	0.40		0.36		0.41		0.14		0.79	

* Median of differences in miRNA levels between BC tissue and normal adjacent tissue (control) samples; the results were normalized to the control. Significant differences are highlighted in bold.

**Table 4:** Association of miR-19b, miR-22, miR-222, miR-378a and miR-181a expression levels with clinicopathologic characteristics of ER- and/or PR-positive BC and ER-and-PR-negative BC.

Characteristics		*n*	Relative Level * of miRNA and *p*-Value									
			miR-19b	*p*-Value	miR-22	*p*-Value	miR-222	*p*-Value	miR-378a	*p*-Value	miR-181a	*p*-Value
ER^+^ and/or PR^+^												
T stage	T1	46	0.80	0.088	0.85	0.124	0.92	**0.009**	0.27	0.152	1.22	0.332
	T2–T4	84	0.36		0.39		0.29		0.18		0.80	
N stage	N0	70	0.58	0.173	0.70	0.301	0.44	0.241	0.24	0.593	1.20	0.492
	N1–N3	60	0.37		0.38		0.43		0.18		0.80	
Ki-67 index (%)	<M **	66	0.52	0.507	0.62	0.176	0.43	0.308	0.18	0.817	0.77	0.551
	≥M **	64	0.38		0.34		0.40		0.22		1.03	
ER score	6–8	113	0.47	0.960	0.49	0.872	0.32	0.477	0.19	0.968	0.82	0.577
	0–5	17	0.65		0.40		0.28		0.12		0.60	
PR score	6–8	74	0.38	0.090	0.48	0.841	0.41	0.819	0.18	0.454	0.77	0.495
	0–5	56	0.58		0.37		0.38		0.24		0.88	
ER^−^ and PR^−^												
T stage	T1	18	0.48	0.858	0.78	0.625	0.60	0.321	0.34	0.808	1.01	0.775
	T2–T4	26	0.43		0.48		0.47		0.20		0.90	
N stage	N0	29	0.46	0.958	0.49	0.903	0.54	0.825	0.23	0.722	1.11	0.445
	N1–N3	15	0.37		0.62		0.64		0.19		0.80	
Ki-67 index (%)	≤M	23	0.51	0.158	0.96	**0.024**	0.71	**0.027**	0.44	**0.035**	1.71	**0.003**
	>M	21	0.28		0.36		0.28		0.10		0.58	

* Median of relative differences in miRNA amounts between breast tumors and paired samples of normal adjoining (control) tissue; the results were normalized to the control. ** M—median value. For ER^−^ and/or PR-positive BC, median = 12, and for ER-and-PR-negative cancer, median = 35. Significant differences are highlighted in bold.

**Table 5:** Association of miR-19b, miR-22, miR-222, miR-378a and miR-181a expression with clinicopathologic characteristics of HER2 1+, 2+, and 3+ tumors and HER2 0 BC.

Characteristics		*n*	Relative Level * of miRNA and *p*-Value									
			miR-19b	*p*-Value	miR-22	*p*-Value	miR-222	*p*-Value	miR-378a	*p*-Value	miR-181a	*p*-Value
HER2 1+, 2+, 3+												
T stage	T1	24	0.83	**0.048**	1.30	**0.037**	1.00	**0.004**	0.63	**0.001**	1.58	**0.044**
	T2–T4	50	0.32		0.43		0.28		0.19		0.80	
N stage	N0	38	0.34	0.827	0.45	0.268	0.39	0.885	0.27	0.339	1.33	0.416
	N1–N3	36	0.39		0.58		0.70		0.34		0.80	
PR score	6–8	27	0.37	0.271	0.50	0.889	0.32	0.527	0.19	0.317	0.72	**0.037**
	0–5	47	0.48		0.47		0.59		0.35		1.48	
HER2 score	2–3	43	0.57	0.424	0.55	0.316	0.78	**0.029**	0.47	**0.003**	1.46	**0.019**
	1	31	0.34		0.50		0.32		0.18		0.66	
HER2 0												
T stage	T1	40	0.48	0.610	0.57	0.522	0.51	0.156	0.15	0.540	0.66	0.926
	T2–T4	60	0.49		0.38		0.41		0.18		0.91	
N stage	N0	61	0.60	**0.046**	0.85	**0.008**	0.51	0.127	0.21	0.075	0.98	0.515
	N1–N3	39	0.34		0.30		0.41		0.13		0.66	
PR score	6–8	47	0.41	0.197	0.41	0.884	0.42	0.710	0.13	0.219	0.85	0.875
	0–5	53	0.54		0.42		0.45		0.18		0.65	
HER2 0, ER^+^/PR^+^												
T stage	T1	31	0.45	0.706	0.47	0.800	0.41	0.138	0.20	0.771	0.69	0.962
	T2–T4	49	0.46		0.36		0.28		0.13		0.85	
N stage	N0	45	0.61	**0.034**	0.85	**0.009**	0.40	0.144	0.24	0.108	0.74	0.677
	N1–N3	35	0.32		0.20		0.27		0.11		0.75	
PR score	6–8	47	0.41	0.342	0.41	0.504	0.42	0.504	0.13	0.550	0.85	0.355
	0–5	33	0.52		0.35		0.28		0.19		0.64	

* Medians of relative differences in miRNA levels between breast tumors and paired samples of normal adjoining (control) tissue; the results were normalized to the control. Significant differences are highlighted in bold.

**Table 6:** Association of miR-19b, miR-22, miR-222, miR-378a and miR-181a expression levels with clinicopathologic characteristics of HER2-overexpressing/amplified tumors and ER+, PR+, HER2 0, 1+ BC.

Characteristics		*n*	Relative Level * of miRNA and *p*-Value									
			miR-19b	*p*-Value	miR-22	*p*-Value	miR-222	*p*-Value	miR-378a	*p*-Value	miR-181a	*p*-Value
HER2 2+, 3+												
T stage	T1	16	0.81	0.479	1.09	0.434	1.00	**0.021**	0.62	**0.033**	1.55	0.761
	T2–T4	27	0.37		0.44		0.29		0.25		1.20	
N stage	N0	27	0.33	0.161	0.45	0.199	0.68	0.372	0.41	0.118	1.40	0.736
	N1–N3	16	0.88		1.05		0.93		0.79		1.64	
HER2 0, 1+, ER^+^ and/or PR^+^												
T stage	T1	48	0.48	0.379	0.60	0.745	0.51	0.094	0.16	0.993	0.73	0.610
	T2–T4	83	0.40		0.39		0.32		0.18		0.77	
N stage	N0	72	0.56	**0.028**	0.67	0.090	0.43	0.131	0.19	0.292	0.77	0.680
	N1–N3	59	0.31		0.36		0.34		0.15		0.66	

* Medians of relative differences in miRNA levels between breast tumors and paired samples of normal adjoining (control) tissue; the results were normalized to the control. Significant differences are highlighted in bold.

**Table 7:** Association of miR-19b, miR-22, miR-222, miR-378a and miR-181a expression with clinicopathologic characteristics of breast tumors with the Ki-67 index < 14% or ≥ 14.

Characteristics		*n*	Relative Level * of miRNA and *p*-Value									
			miR-19b	*p*-Value	miR-22	*p*-Value	miR-222	*p*-Value	miR-378a	*p*-Value	miR-181a	*p*-Value
Ki-67 < 14%												
T stage	T1	26	0.81	0.334	0.87	0.665	0.54	0.199	0.27	0.696	0.92	0.966
	T2–T4	54	0.46		0.54		0.46		0.19		0.88	
N stage	N0	45	0.59	0.106	0.88	0.273	0.45	0.536	0.26	0.458	1.25	0.995
	N1–N3	35	0.42		0.58		0.46		0.19		0.83	
PR score	6–8	46	0.50	0.738	0.75	0.325	0.62	0.319	0.20	0.458	0.93	0.300
	0–5	34	0.51		0.44		0.38		0.25		0.78	
Ki-67 ≥ 14%												
T stage	T1	38	0.51	0.372	0.57	0.068	0.84	**0.006**	0.32	0.087	1.11	0.158
	T2–T4	56	0.31		0.30		0.28		0.16		0.77	
N stage	N0	54	0.45	0.539	0.49	0.539	0.48	0.443	0.21	0.780	1.15	0.228
	N1–N3	40	0.32		0.34		0.45		0.16		0.64	
PR score	6–8	28	0.24	**0.017**	0.16	**0.046**	0.21	**0.022**	0.09	**0.011**	0.37	**0.047**
	0–5	66	0.54		0.46		0.55		0.23		0.98	

* Medians of relative differences in miRNA amounts between breast tumors and samples of paired normal adjoining (control) tissue; the results were normalized to the control. Significant differences are highlighted in bold.

**Table 8:** The identified relation between differences in miRNA expression and tumor characteristics.

Observed Change	Associated Tumor Characteristics				
	ER^+^ and/or PR^+^	ER^−^ and PR^−^	HER2^+^	HER2 0, ER^+^ and/or PR^+^	Ki-67 ≥ 14%
miR-19b decreased				N1–N3 stages	PR score 6–8
miR-22 decreased		Ki-67 > 35%		N1–N3 stages	PR score 6–8
miR-222 decreased	stages T2–T4	Ki-67 > 35%	stages T2–T4 HER2 score 1		T2–T4 stages PR score 6–8
miR-378a decreased		Ki-67 > 35%	stages T2–T4 HER2 score 1		PR score 6–8
miR-181a decreased		Ki-67 > 35%	HER2 score 1		PR score 6–8

## Data Availability

The data presented in this study are available on request from the corresponding author.

## References

[1] Nagai H., Kim Y.H. (2017). Cancer prevention from the perspective of global cancer burden patterns. J. Thorac. Dis..

[2] Shah R., Rosso K., Nathanson S.D. (2014). Pathogenesis, prevention, diagnosis and treatment of breast cancer. World J. Clin. Oncol..

[3] Cicatiello L., Addeo R., Sasso A., Altucci L., Petrizzi V.B., Borgo R., Cancemi M., Caporali S., Caristi S., Scafoglio C. (2004). Estrogens and progesterone promote persistent CCND1 gene activation during G1 by inducing transcriptional derepression via c-Jun/c-Fos/estrogen receptor (progesterone receptor) complex assembly to a distal regulatory element and recruitment of cyclin D1 to its own gene promoter. Mol. Cell. Biol..

[4] Hyder S.M. (2002). The role of steroid hormones on the regulation of vascular endothelial growth factor. Am. J. Pathol..

[5] Wang C., Mayer J.A., Mazumdar A., Fertuck K., Kim H., Brown M., Brown P.H. (2011). Estrogen induces c-myc gene expression via an upstream enhancer activated by the estrogen receptor and the AP-1 transcription factor. Mol. Endocrinol..

[6] Meng X., Song S., Jiang Z.F., Sun B., Wang T., Zhang S.h., Wu S.h. (2016). Receptor conversion in metastatic breast cancer: A prognosticator of survival. Oncotarget.

[7] Hua H., Zhang H., Kong Q., Jiang Y. (2018). Mechanisms for estrogen receptor expression in human cancer. Exp. Hematol. Oncol..

[8] Harvey J.M., Clark G.M., Osborne C.K., Allred D.C. (1999). Estrogen receptor status by immunohistochemistry is superior to the ligand-binding assay for predicting response to adjuvant endocrine therapy in breast cancer. J. Clin. Oncol..

[9] Kalinina T.S., Kononchuk V.V., Yakovleva A.K., Alekseenok E.Y., Sidorov S.V., Gulyaeva L.F. (2020). Association between lymph node status and expression levels of androgen receptor, miR-185, miR-205, and miR-21 in breast cancer subtypes. Int. J. Breast Cancer.

[10] Chen C., Ridzon D.A., Broomer A.J., Zhou Z., Lee D.H., Nguyen J.T., Barbisin M., Xu N.L., Mahuvakar V.R., Andersen M.R. (2005). Real-time quantification of microRNAs by stem-loop RT-PCR. Nucleic Acids Res..

[11] Huang H.Y., Lin Y.C., Li J., Huang K.Y., Shrestha S., Hong H.C., Tang Y., Chen Y.G., Jin C.N., Yu Y. (2020). miRTarBase 2020: Updates to the experimentally validated microRNA-target interaction database. Nucleic Acids Res..

[12] Gilam A., Shai A., Ashkenazi I., Sarid L.A., Drobot A., Bickel A., Shomron N. (2017). MicroRNA regulation of progesterone receptor in breast cancer. Oncotarget.

[13] Wang B., Li J., Sun M., Sun L., Zhang X. (2014). miRNA expression in breast cancer varies with lymph node metastasis and other clinicopathologic features. IUBMB Life.

[14] Ikeda K., Horie-Inoue K., Ueno T., Suzuki T., Sato W., Shigekawa T., Osaki A., Saeki T., Berezikov E., Mano H. (2015). miR-378a-3p modulates tamoxifen sensitivity in breast cancer MCF-7 cells through targeting GOLT1A. Sci. Rep..

[15] Xiong J., Yu D., Wei N., Fu H., Cai T., Huang Y., Wu C., Zheng X., Du Q., Lin D. (2010). An estrogen receptor alpha suppressor, microRNA-22, is downregulated in estrogen receptor alpha-positive human breast cancer cell lines and clinical samples. FEBS J..

[16] Zou Q., Tang Q., Pan Y., Wang X., Dong X., Liang Z., Huang D. (2017). MicroRNA-22 inhibits cell growth and metastasis in breast cancer via targeting of SIRT1. Exp. Ther. Med..

[17] Stinson S., Lackner M.R., Adai A.T., Yu N., Kim H.J., O’Brien C., Spoerke J., Jhunjhunwala S., Boyd Z., Januario T. (2011). TRPS1 targeting by miR-221/222 promotes the epithelial-to-mesenchymal transition in breast cancer. Sci. Signal..

[18] Chernyy V., Pustylnyak V., Kozlov V., Gulyaeva L. (2018). Increased expression of miR-155 and miR-222 is associated with lymph node positive status. J. Cancer.

[19] Han S.H., Kim H.J., Gwak J.M., Kim M., Chung Y.R., Park S.Y. (2017). MicroRNA-222 Expression as a predictive marker for tumor progression in hormone receptor-positive breast cancer. J. Breast Cancer.

[20] Yoshimoto N., Toyama T., Takahashi S., Sugiura H., Endo Y., Iwasa M., Fujii Y., Yamashita H. (2011). Distinct expressions of microRNAs that directly target estrogen receptor α in human breast cancer. Breast Cancer Res. Treat..

[21] Deng L., Lei Q., Wang Y., Wang Z., Xie G., Zhong X., Wang Y., Chen N., Qiu Y., Pu T. (2017). Downregulation of miR-221-3p and upregulation of its target gene PARP1 are prognostic biomarkers for triple negative breast cancer patients and associated with poor prognosis. Oncotarget.

[22] Wu Q., Guo L., Jiang F., Li L., Li Z., Chen F. (2015). Analysis of the miRNA-mRNA-lncRNA networks in ER+ and ER- breast cancer cell lines. J. Cell. Mol. Med..

[23] Li C., Zhang J., Ma Z., Zhang F., Yu W. (2018). miR-19b serves as a prognostic biomarker of breast cancer and promotes tumor progression through PI3K/AKT signaling pathway. Onco Targets Ther..

[24] Yin R., Guo L., Gu J., Li C., Zhang W. (2018). Overexpressing miR-19b-1 suppress breast cancer growth by inhibiting tumor microenvironment induced angiogenesis. Int. J. Biochem. Cell Biol..

[25] Anfossi S., Giordano A., Gao H., Cohen E.N., Tin S., Wu Q., Garza R.J., Debeb B.G., Alvarez R.H., Valero V. (2014). High serum miR-19a levels are associated with inflammatory breast cancer and are predictive of favorable clinical outcome in patients with metastatic HER2+ inflammatory breast cancer. PLoS ONE.

[26] Kondo N., Toyama T., Sugiura H., Fujii Y., Yamashita H. (2008). miR-206 Expression is down-regulated in estrogen receptor alpha-positive human breast cancer. Cancer Res..

[27] Li Y., Hong F., Yu Z. (2013). Decreased expression of microRNA-206 in breast cancer and its association with disease characteristics and patient survival. J. Int. Med. Res..

[28] Ahmad A., Ginnebaugh K.R., Sethi S., Chen W., Ali R., Mittal S., Sarkar F.H. (2015). miR-20b is up-regulated in brain metastases from primary breast cancers. Oncotarget.

[29] Castellano L., Giamas G., Jacob J., Coombes R.C., Lucchesi W., Thiruchelvam P., Barton G., Jiao L.R., Wait R., Waxman J. (2009). The estrogen receptor-alpha-induced microRNA signature regulates itself and its transcriptional response. Proc. Natl. Acad. Sci. USA.

[30] Luengo-Gil G., García-Martínez E., Chaves-Benito A., Conesa-Zamora P., Navarro-Manzano E., González-Billalabeitia E., García-Garre E., Martínez-Carrasco A., Vicente V., Ayala de la Peña F. (2019). Clinical and biological impact of miR-18a expression in breast cancer after neoadjuvant chemotherapy. Cell Oncol..

[31] Tokar T., Pastrello C., Rossos A.E.M., Abovsky M., Hauschild A.C., Tsay M., Lu R., Jurisica I. (2018). mirDIP 4.1-integrative database of human microRNA target predictions. Nucleic Acids Res..

[32] Pandey D.P., Picard D. (2009). miR-22 inhibits estrogen signaling by directly targeting the estrogen receptor alpha mRNA. Mol. Cell. Biol..

[33] Zhao J.J., Lin J., Yang H., Kong W., He L., Ma X., Coppola D., Cheng J.Q. (2008). MicroRNA-221/222 negatively regulates estrogen receptor alpha and is associated with tamoxifen resistance in breast cancer. J. Biol. Chem..

[34] Panda H., Chuang T.D., Luo X., Chegini N. (2012). Endometrial miR-181a and miR-98 expression is altered during transition from normal into cancerous state and target PGR, PGRMC1, CYP19A1, DDX3X, and TIMP3. J. Clin. Endocrinol. Metab..

[35] Toms D., Xu S., Pan B., Wu D., Li J. (2015). Progesterone receptor expression in granulosa cells is suppressed by microRNA-378-3p. Mol. Cell. Endocrinol..

[36] Lambein K., Van Bockstal M., Vandemaele L., Geenen S., Rottiers I., Nuyts A., Matthys B., Praet M., Denys H., Libbrecht L. (2013). Distinguishing score 0 from score 1+ in HER2 immunohistochemistry-negative breast cancer: Clinical and pathobiological relevance. Am. J. Clin. Pathol..

[37] Schettini F., Chic N., Brasó-Maristany F., Paré L., Pascual T., Conte B., Martínez-Sáez O., Adamo B., Vidal M., Barnadas E. (2021). Clinical, pathological, and PAM50 gene expression features of HER2-low breast cancer. NPJ Breast Cancer.

[38] Peng Y., Croce C.M. (2016). The role of MicroRNAs in human cancer. Signal Transduct. Target. Ther..

[39] Kurozumi S., Yamaguchi Y., Kurosumi M., Ohira M., Matsumoto H., Horiguchi J. (2017). Recent trends in microRNA research into breast cancer with particular focus on the associations between microRNAs and intrinsic subtypes. J. Hum. Genet..

[40] Chen B., Tang H., Liu X., Liu P., Yang L., Xie X., Ye F., Song C., Xie X., Wei W. (2015). miR-22 as a prognostic factor targets glucose transporter protein type 1 in breast cancer. Cancer Lett..

[41] Gold E.B. (2011). The timing of the age at which natural menopause occurs. Obstet. Gynecol. Clin. North. Am..

[42] Zong Y., Zhang Y., Sun X., Xu T., Cheng X., Qin Y. (2019). miR-221/222 promote tumor growth and suppress apoptosis by targeting lncRNA GAS5 in breast cancer. Biosci. Rep..

[43] Agarwal V., Bell G.W., Nam J.W., Bartel D.P. (2015). Predicting effective microRNA target sites in mammalian mRNAs. Elife.

[44] Maleki E., Ghaedi K., Shahanipoor K., Karimi Kurdistani Z. (2018). Down-regulation of microRNA-19b in hormone receptor-positive/HER2-negative breast cancer. APMIS.

[45] Yang C., Tabatabaei S.N., Ruan X., Hardy P. (2017). The dual regulatory role of miR-181a in breast cancer. Cell. Physiol. Biochem..

[46] Li J.Y., Ou Z.L., Yu S.J., Gu X.L., Yang C., Chen A.X., Di G.H., Shen Z.Z., Shao Z.M. (2012). The chemokine receptor CCR4 promotes tumor growth and lung metastasis in breast cancer. Breast Cancer Res. Treat..

[47] Donnelly S.M., Paplomata E., Peake B.M., Sanabria E., Chen Z., Nahta R. (2014). P38 MAPK contributes to resistance and invasiveness of HER2- overexpressing breast cancer. Curr. Med. Chem..

[48] Kirouac D.C., Du J., Lahdenranta J., Onsum M.D., Nielsen U.B., Schoeberl B., McDonagh C.F. (2016). HER2+ Cancer Cell Dependence on PI3K vs. MAPK Signaling Axes Is Determined by Expression of EGFR, ERBB3 and CDKN1B. PLoS Comput. Biol..

[49] O’Sullivan C.C., Suman V.J., Goetz M.P. (2019). The emerging role of CDK4/6i in HER2-positive breast cancer. Ther. Adv. Med. Oncol..

[50] Eichner L.J., Perry M.C., Dufour C.R., Bertos N., Park M., St-Pierre J., Giguère V. (2010). miR-378(∗) mediates metabolic shift in breast cancer cells via the PGC-1β/ERRγ transcriptional pathway. Cell Metab..

